# Loss of Competition in the Outside Host Environment Generates Outbreaks of Environmental Opportunist Pathogens

**DOI:** 10.1371/journal.pone.0071621

**Published:** 2013-08-16

**Authors:** Jani Anttila, Lasse Ruokolainen, Veijo Kaitala, Jouni Laakso

**Affiliations:** Integrative Ecology Unit, Department of Biosciences, University of Helsinki, Helsinki, Finland; Louisiana State University, United States of America

## Abstract

Environmentally transmitted pathogens face ecological interactions (e.g., competition, predation, parasitism) in the outside-host environment and host immune system during infection. Despite the ubiquitousness of environmental opportunist pathogens, traditional epidemiology focuses on obligatory pathogens incapable of environmental growth. Here we ask how competitive interactions in the outside-host environment affect the dynamics of an opportunist pathogen. We present a model coupling the classical SI and Lotka–Volterra competition models. In this model we compare a linear infectivity response and a sigmoidal infectivity response. An important assumption is that pathogen virulence is traded off with competitive ability in the environment. Removing this trade-off easily results in host extinction. The sigmoidal response is associated with catastrophic appearances of disease outbreaks when outside-host species richness, or overall competition pressure, decreases. This indicates that alleviating outside-host competition with antibacterial substances that also target the competitors can have unexpected outcomes by providing benefits for opportunist pathogens. These findings may help in developing alternative ways of controlling environmental opportunist pathogens.

## Introduction

Traditionally, ecological and eco-evolutionary epidemiological models describe the dynamics of infectious diseases by considering susceptible, infected and recovered hosts with host-to-host, or host-environment-host transmission [1]–[5]. A number of modifications–such as seasonality [6], or within-host dynamics [7]–have been introduced to the SI- and SIR-models in various attempts to explain recurrent outbreak disease dynamics. However, natural epidemics often show a variety of dynamics that do not correspond to the predictions made by the classical models. One reason for this is that the underlying assumptions on disease transmission are unrealistic for pathogens that spend a considerable amount, or even the most part of their life cycle, in the outside-host environment. A large proportion of opportunist pathogen species also grow actively in the outside-host environment. These environmental pathogens form an increasing problem for human health [8], and thus a better theoretical understanding of their epidemiology is required. Currently, most models for environmental transmission allow only decay of pathogens in the outside-host environment [9], [10,], but see [11]. In addition, the outside-host environment is riddled with other microbes which frequently interact with the pathogen. This means that while active growth as well as ecological interactions in the environment are likely to be profoundly important, they are yet poorly understood factors in disease dynamics.

The role of environmental transmission in disease dynamics and the evolution of virulence has attracted increasing interest [10], [12], both due to human pathogen outbreaks such as cholera [13] and emergent animal diseases, e.g. columnaris disease [14]. Worldwide, there is an ongoing battle against opportunistic infections, which are often persistent due to the pathogens’ ability to grow outside hosts. Broad-spectrum antibiotics and disinfectants are used *en masse* to prevent environmental infections to humans and cultivated animals. This is likely to cause changes in the composition of environmental communities and have an impact on ecosystem functioning, health and disease [15]. Theoretically, an environmentally transmitted pathogen can be highly lethal as the trade-offs between transmission and virulence associated with obligate pathogens are reduced [16]. This is because by killing a host–which is not required to be alive for pathogen transmission–the pathogen gains access to an enormously rich resource for saprotrophic growth, which can lead to a positive transmission-virulence relationship [17]. In most studies, however, the environment represents simply a reservoir into which the pathogen particles are shed from infected hosts and from which the surviving pathogen individuals may re-enter susceptible hosts, without explicit description of the outside-host dynamics other than the decay rate of the pathogen.

Interspecific interactions, such as competition, mutualism, predation, and parasitism constitute the core of ecological research. An important implication of these interactions is that the dynamics and stability of individual populations within ecological networks (e.g., communities or food webs) can strongly depend on the composition of these networks and the details of between-species interactions [18]–[20]. In general, similar co-occurring species compete for limiting resources [21] and are attacked by parasites and predators [22]. All of these different ecological interactions can affect the density of pathogens and other interacting species in the community, thereby affecting the probabilities of infection outbreaks. Therefore, understanding the role of ecological interactions in the outside-host environment is likely to be of great importance for uncovering mechanisms behind the dynamics of many environmentally transmitted diseases such as *Vibrio cholera* [23], group A *streptococci* [24], *Staphylococcus aureus* [25], and *Flavobacterium columnare* [14].

We explore the dynamics of a model that combines environmental opportunist pathogen–host dynamics to community dynamics outside the host. By the term environmental opportunist pathogen we mean an organism that is both (i) able to grow in the environment in the absence of hosts and (ii) infect susceptible hosts. Whether the pathogen can infect one or several host species is not important in this simple model in which we consider all (susceptible) hosts similar from the pathogens perspective. The model contains susceptible and infected hosts as in a classical SI-model and a competitive community in the outside-host environment. One of the competitors is a pathogen that can return from a dead host to the environment and thus the host does not represent an ecological or evolutionary dead end for the pathogen. This is opposite to the common assumption of the theory of “co-incidental virulence” [26]. Further, we assume that there is a trade-off between virulence and environmental competitive ability. This assumption also differs from the expectation under co-incidental virulence theory [26], where resource acquisition or fighting against natural enemies outside the host are positively linked to a pathogens ability to cause infections. Life-history trade-offs can reduce virulence because the machineries for resource acquisition and defence in the outside- vs. inside-host environments require specialisation [27], [28]. The choice of the functional form of the infectivity response can crucially affect the model dynamics [29], [30]. We explore both linear and sigmoidal infection rate in response to pathogen density, and assume that the infected hosts can either recover back to the susceptible class or die from the disease. The sigmoidal infectivity response incorporates dose-dependence, i.e., exposure to pathogen densities below a certain level is unlikely to cause an infection, as observed in many laboratory experiments [31]–[34]. The model behavior is explored in a parameter range that is likely to cover typical environmentally growing opportunist micro-parasites (e.g., bacteria, protozoa, or fungi) that infect multicellular hosts ranging from taxa with fast growth rates (e.g., nematodes and insects) to taxa with slow growth rates (e.g., vertebrates).

A striking feature of the model is that reducing competitor species richness in the outside-host environment can lead to an abrupt emergence of disease outbreaks. If the infectivity response of the pathogen is a sigmoidal function of pathogen density in the environment, epidemiological dynamics are sensitive to the intensity of competitive suppression by the outside-host community. With linear infectivity response and pathogen growth in the environment no disease outbreaks are observed, i.e. population dynamics remain stable, and pathogen and host densities are relatively insensitive to manipulation of diversity. Under sigmoidal infectivity, reduction of competitive pressure on the pathogen, either due to loss of diversity from the outside-host community (e.g., due to use of disinfectants), or increased loss rate of all species in the outside-host community (e.g., due to application of non-specific antibiotics) can lead to catastrophic disease outbreaks.

## Methods

The dynamical model is a combination of the classical SI-disease dynamics [1] for hosts and the Lotka–Volterra competition model for an outside-host community. The susceptible and infected hosts at time *t* are denoted by *S*(*t*) and *I*(*t*), respectively. The pathogen, *P*(*t*), has *n* competitors, with densities denoted by *B_i_*(*t*), i.e., community size is *N* = *n* +1. The population densities vary according to the following differential equations:
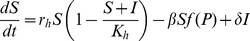
(1)

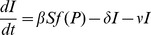
(1)

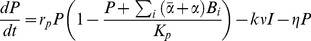
(1)

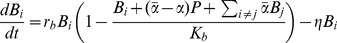
(1)


In the absence of an infection, susceptible hosts follow a logistic growth model with a growth rate *r_h_* and a carrying capacity *K_h_* (eqn. 1.1). In the presence of an infective pathogen infected host individuals are formed. The infected individuals compete for resources with the susceptible individuals, but do not contribute to host reproduction (this assumption has no qualitative effect on the dynamics). Susceptible hosts are infected with a rate *βSf*(*P*) depending on the infectivity response *f*(*P*). Two alternative infectivity response functions were explored. Most previous theoretical work has assumed a linear infectivity response. However, we argue that a more realistic assumption for many circumstances is a sigmoidal dose-dependent response that is supported by empirical data [31]–[34] as well as theoretical analysis [35]. The sigmoidal infectivity function has the following mechanistic interpretation. With low pathogen densities the immune system can effectively overcome most pathogen invasions and therefore the probability of an infection per unit time must increase nonlinearly at low densities. With high pathogen densities the effect of increasing pathogen density on the probability of infection per time unit must saturate since the development of an infection is not an instantaneous process. This type of functional response has been studied by Regoes et al. [36] for direct transmission in the context of the classical SIR-model. Mechanisms behind sigmoidal infectivity response can also include saturation of the immune system with a large number of invaders, density-dependent accumulation of enzymes that allow breaching the immune system or expression of virulence factors due to density-dependent bacterial communication, i.e., quorum sensing [37]. A Hill function (eqn. 2) was chosen as the functional form for the sigmoid infectivity response *f*(*P*), because it is a simple way to conceive this type of dose-dependence for infections [36]:
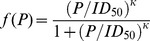
(2)where parameters *ID*_50_ and *κ* affects the shape of the response. Eqn. 2 is used as a convenience function with no particular mechanistic underpinning. A classical linear term with *f*(*P*)* = P* was used for comparison. In either case, the infectivity rate is scaled by parameter *β* in eqn (1.1). In eqn. (1.2) infected hosts die from the disease with rate *ν* and recover from the infection with rate *δ*. With the parameter set in Table 1, every 3/7:th infection leads to host death. In case of recovery the pathogens inside a host are killed by the host immune system.

**Table 1:** Model parameters and values used in the model.

Parameter	Interpretation	Values used in simulations
*n*	Number of competitors in the environment	0–17
*r_h_*	Host growth rate	0.1
*r_p_*	Pathogen growth rate	2.0
*r_b_*	Competitor growth rate	2.0
*K_h_*	Host carrying capacity	100
*K_p_*	Outside host pathogen carrying capacity	10000
*K_b_*	Outside host community carrying capacity	10000
*ID*_50_	Infectious dose at which 50% of hosts are infected.	3150
*κ*	Slope parameter of the sigmoid infectivity function	4
*β*	Maximum infectivity	4
*δ*	Host recovery rate from infection	0.6
*ν*	Infection kill rate	0.1
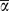	Between species competition strength	0.5
*α*	Competitive ability reduction	0.05, 0–0.15
*k*	Number of pathogens released at host death	500
*η*	Pathogen mortality	1.0, 0–2.5

The parameters are chosen to represent a realistic scenario with a bacterial pathogen and a small multi-cellular host (see text).

The pathogen (*P*) and the competing non-pathogenic strains (*B_i_*) grow logistically with rates *r_p_* and *r_b_*. Carrying capacities are *K_p_* and *K_b_* in a Lotka–Volterra competition setting, respectively (for simplicity, *r_p_* = *r_b_*, and *K_p_* = *K_b_*). The competition in the outside-host community was modeled with a very general diffuse competition model where the competitive ability of non-pathogens against the pathogen was varied. The intraspecific competition coefficients were set to 1, whereas between species interaction strength is given by 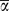 (eqns. 1.3, 1.4; here set 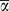 = 0.5). It is assumed that the pathogen pays a cost of its ability to cause infections (e.g., due to extra biochemical machinery) in form of reduced competitive ability. This is realised as competition against the pathogen equal to 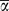
+α, and for the pathogen in competition with other species 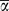 – α, with α being the reduction in pathogen competitive ability. A diffuse competition model is used as a simplification in this model. This is justified since generating between species coefficients randomly with a mean 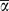 produces, on average, the same dynamics. In the case of positive correlation between virulence and competitive ability, i.e. negative α, a trivial result in our model is that the host is driven to extinction by the pathogen.

A mortality term with rate *η* was included for scenarios where growth is not only self-limited but there is another density-independent mortality factor that removes bacteria from the system (e.g., antibacterial substances, or physical outflow from the system). If hosts are sparse, intense outside-host competition in addition to out-flow mortality *η* can drive the pathogen extinct while waiting for the next infection. The effect competition has on the pathogen species could be realised either through reduction in pathogen competitive ability (α) or increasing the number of competitors present in the outside host community (*n*). The equilibrium pathogen density reaches zero with *α = *1/(2*n*) in the absence of the host, due to competition. The survival of the pathogen beyond this level of competition is still possible via coupling to host dynamics due to fitness benefits gained by inside-hosts growth. On the other hand if the pathogen is very infective and lethal, it may kill all the suitable hosts and thereafter become extinct by competitive exclusion. This outcome resembles that of the classical trade-off between virulence and transmission, albeit via an entirely different mechanism. Equilibrium densities for the pathogen and non-pathogenic species in the absence of hosts are:
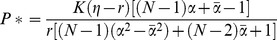
(3)

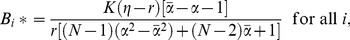
(3)where *r* is the common growth rate for all species. Note that P* is only positive when 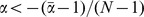 and *r*>*η.*

The model assumes that a burst of pathogens is released to the environment with the death of an infected host. This identifies to saprotrophy where the dead host body is consumed to some degree. As the dead host typically represents an extremely rich resource in comparison to the typical outside-host environment [17], the number of pathogens released from the host can be extremely high. Thus the large flow of pathogens to the environment from the host can in turn lead to a rapid cascade of infections and host deaths that can ultimately result in host extinction [38]. The parameter ranges used in the simulations are given in Table 1.

The pathogen growth rate of two divisions per day represents the lower end of bacterial growth rates. The infected hosts remain infected for a relatively long period since both infection kill rate and recovery rate are low. This is plausible e.g. for an untreated bacterial disease many of which are very persistent. The host growth rate is suitable for a fish host [11].

Outside host community densities are scaled down to make the numbers comparable in order of magnitude to that of hosts. Multiplying outside host carrying capacity and the number of pathogen units released from a dead host by 10^6^ leads to a scenario where the unit of area could be a cubic metre of water with 100 small fish hosts and 10^10^ bacterial cells. The infectivity parameter values used here allow for effective infecting without driving the host extinct too easily. Extensive simulations with randomly selected parameter values indicate that the results presented here are qualitatively robust (Materials S1, Fig. S2, S3).

The model (eq. 1) behaviour was analysed numerically using a Runge-Kutta fourth order routine. Means, maxima, and standard deviations of population densities were recorded from the final 100 time units from each simulation in order to assess the type of the dynamics (e.g. outbreaks). The analysis focused on the asymptotic behavior of the model and excluded the initial transients. Trajectories were simulated for 200 time units that was a sufficiently long time interval for analysing the asymptotic behaviour of the model in all cases. The initial state in all simulations was *S = *100, *I = *0, *P* and *B_i_* chosen from uniform random distribution between 100 and 1600.

## Results

The choice of the infectivity response function is crucial to the behaviour of the model. Therefore the following results are presented according to this dichotomy between linear and sigmoidal infectivity responses.

**Figure 1:**
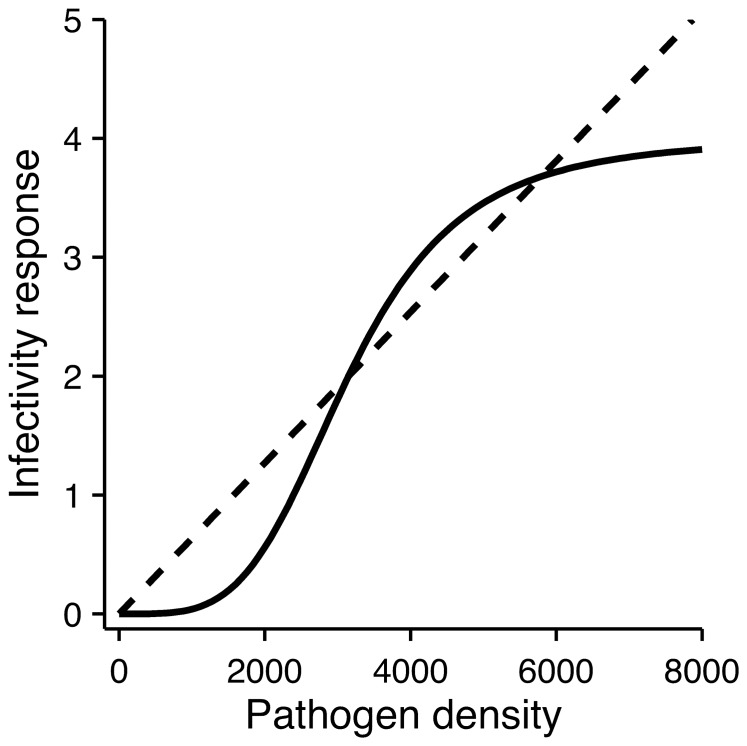
The infectivity response of the pathogen *f*(*P*) is assumed to be either linear (dashed line) or sigmoidal (solid line) function of pathogen density (P). To facilitate comparison, linear infectivity is modeled with rate constant *β*/2*ID*_50_ and the parameters for sigmoidal response (eq. 2) are set to *ID*_50_ = 3150 and *κ* = 4. The curves intersect at *ID*_50_.

**Figure 2:**
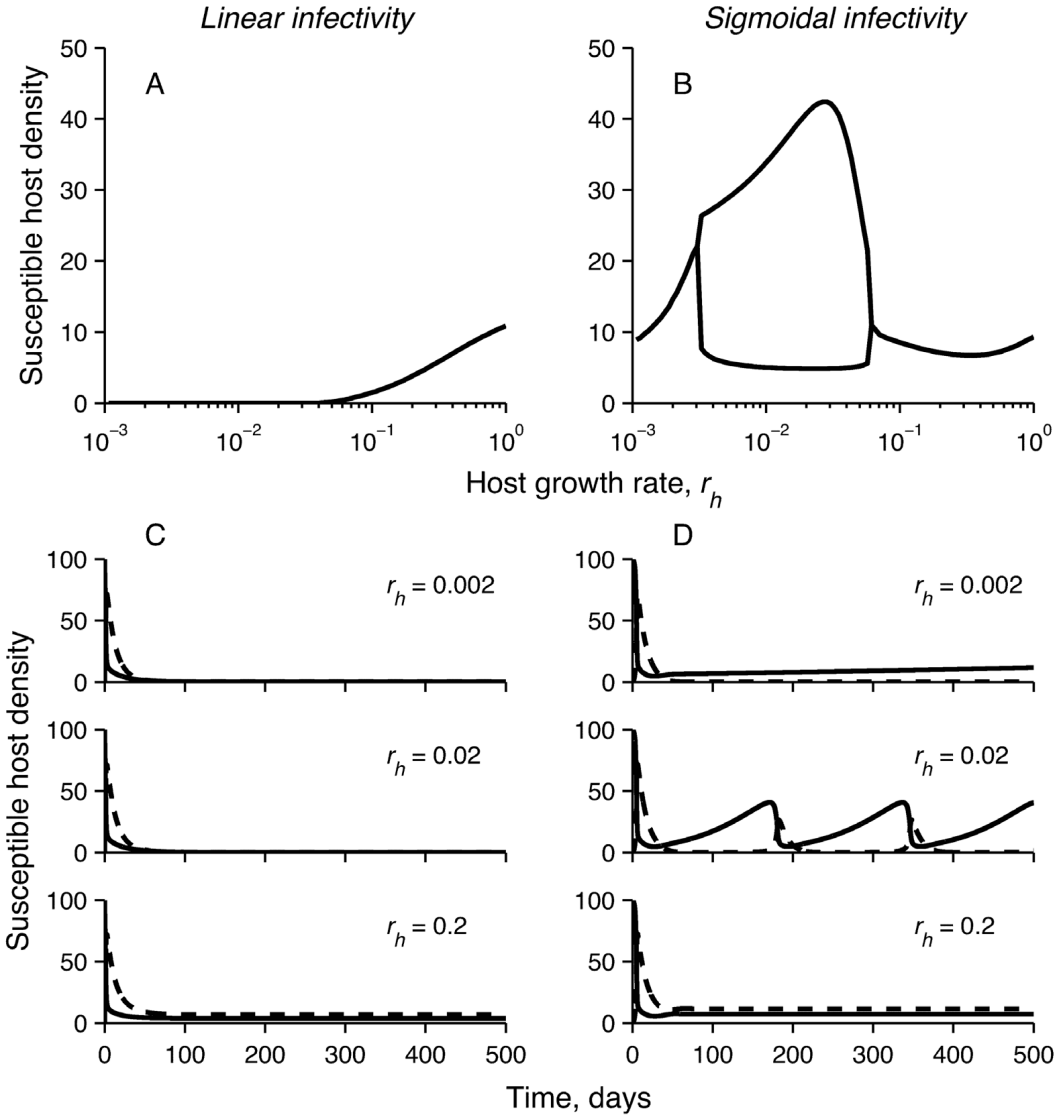
Susceptible host response to increasing host growth rate depending on the form of pathogen infective response (either linear or sigmoidal). Here the competitive disadvantage (α) is fixed to 0.05 and the number of competitor species (*n*) to 6. Linear infectivity response with rate constant *β/*2*ID*_50_ was used in panels (a) and (c), and *ID*_50_ = 3150, *κ* = 4 for sigmoidal response (eqn. 2) in panels (b) and (d).The lines in (a) and (b) represent minima and maxima of population densities. In (c) and (d) the solid line is susceptible host density and the dotted line is infected host density.

**Figure 3:**
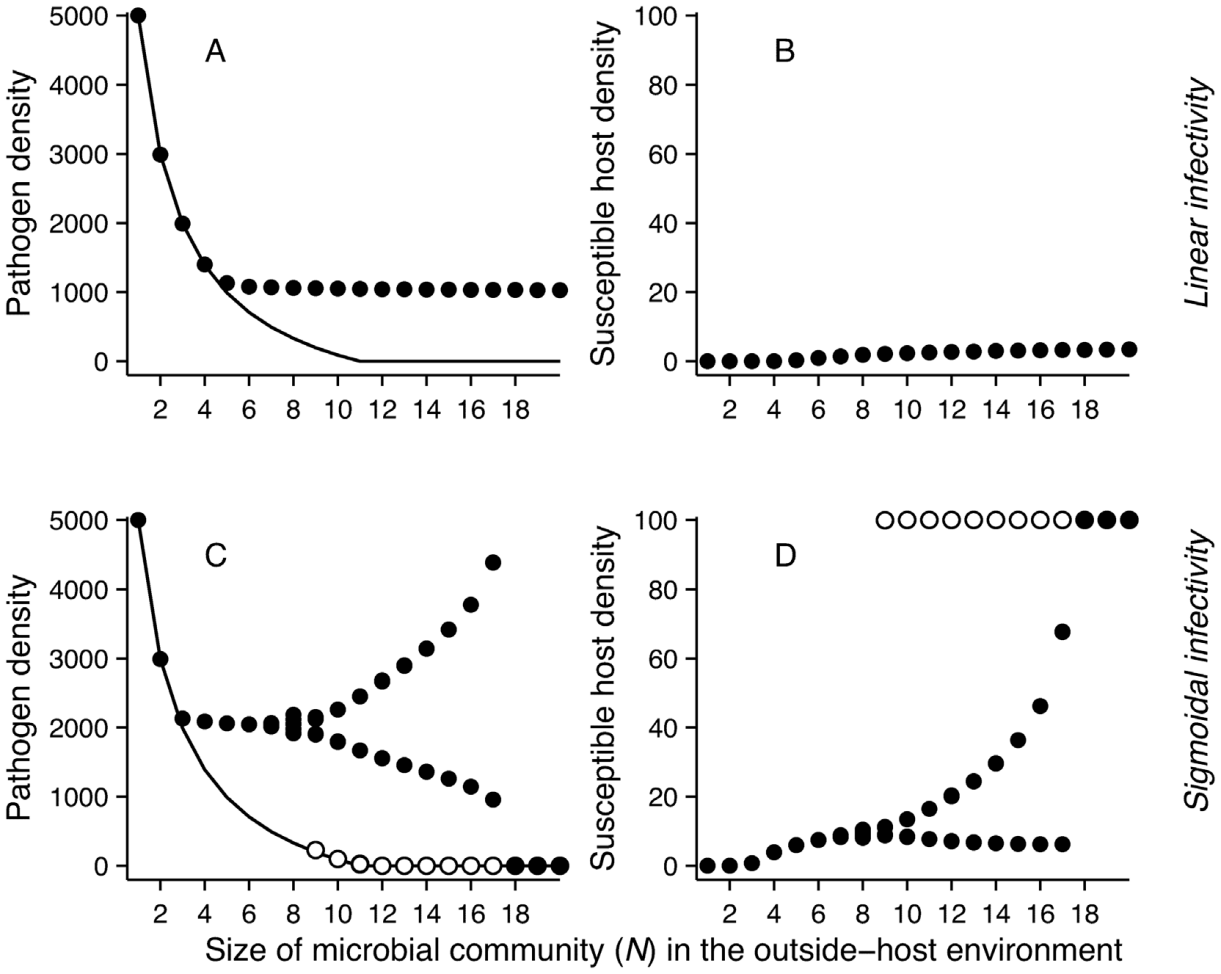
Pathogen and susceptible host response to increasing number of competitors in the outside-host community (*n* = *N* –1). Here α is fixed to 0.05 and linear mortality parameter *η* to 1.0. Linear infectivity response with rate constant *β/*2*ID*_50_ was used in panels (a) and (b), and *ID*_50_ = 3150, *κ* = 4 for sigmoidal response (eqn. 2) in panels (c) and (d). Filled symbols represent minimum and maximum densities. In the cyclic range in (c) and (d) open symbols show the alternative attractor and filled symbols show minima and maxima. The solid line is the equilibrium pathogen density without hosts.

A common assumption in epidemiological models is that there is a linear relationship between pathogen density and the number of infections (i.e., *f*(*P*) = *P*, Fig. 1). The linear response is a benefit to the pathogen since it remains infective even in small doses (Fig. 2a). However, the host is easily driven to extinction unless its growth is sufficiently fast. Under this assumption, increasing outside-host community size is associated with an initial reduction in the density of free-living pathogen, as expected from classical competition theory (Fig. 3a, see e.g., May 1972). Increasing competition in the outside-host community quickly drives the pathogen extinct in the absence of hosts. However, the pathogens ability to infect hosts and use them as resources for reproduction compensates for the reduced competitive ability in the outside-host environment (Fig. 3a). Pathogens persist at a stable density, which is relatively independent of the number of competitors. With the linear response cyclic pathogen dynamics were only observed when pathogens did not grow in the environment (*r_p_*< *η*) and that there was no significant recovery of infected hosts. Linear response stabilises the dynamics because it prevents host supply re-growth by making the pathogen efficient in infecting also when the pathogen density is low. While pathogen infections reduce the number of susceptible hosts well below their carrying capacity (Fig. 3b), competition in the outside-host environment can prevent the pathogen from driving the host extinct.

A pathogen with a sigmoidal transmission differs from the linear transmission in its response to increasing competition pressure in three important ways (Fig. 2, 3c, 3d): (1) Near the point where pathogen growth rate in the absence of hosts approaches zero due to competitive exclusion, increasing outside-host community size gives rise to cyclic pathogen dynamics. (2) After this bifurcation an alternative attractor appears where the pathogen is excluded from the system. This happens because of the Allee effect associated with the sigmoidal dose-response function [36]; if the pathogen is initiated at a sufficiently low density, it is unable to infect susceptible hosts, preventing fitness gains through within-host growth. Also when the initial pathogen density is too high there is a rapid increase in density followed by a drop to very low density after which the pathogen density stays close to zero. (3) Increasing outside-host community size further amplifies the cyclic dynamics on the attractor where the pathogen is present. This continues up to a point where competitive pressure is sufficiently high to prevent pathogen infections independently of initial conditions, leading to an abrupt disappearance of the pathogen from the system.

The range of cyclic dynamics depends on host growth rate (Fig. 2B). Outbreaks arise when the time scale of pathogen release from hosts is comparable to that of host growth rate. If pathogen release happens much faster than hosts grow or recover then no hosts are available for the released pathogen making further cycling impossible. The host growth rate of 0.1 per day used in our simulations is quite high for most large multi-cellular organisms. To represent diseases of slowly growing hosts the infectivity and host-pathogen interaction parameters need to be scaled accordingly to retain the same range of qualitative dynamics. For example cyclic dynamics can be retained at lower host growth rates by lowering infected host recovery and death rates at the same time.

Similar patterns to those shown in Fig. 3, due to increasing size of the outside-host community *N*, can be generated by varying the competitive disadvantage of the pathogen, α, for this parameter set (Fig. S1). This is easy to understand, as increasing either the number of competitors (*n*) or competitive disadvantage (α) decreases the equilibrium density of the pathogen (eqn. 3). Under sigmoidal transmission this means that when the pathogen is unable to survive in the outside-host environment without fitness gains from host infection, the presence of susceptible hosts can be associated with two alternative attractors, with pathogens either present or absent. When competition is intensive enough, pathogens are unable to reach sufficiently high densities for infections to arise, inevitably leading to pathogen extinction.

**Figure 4:**
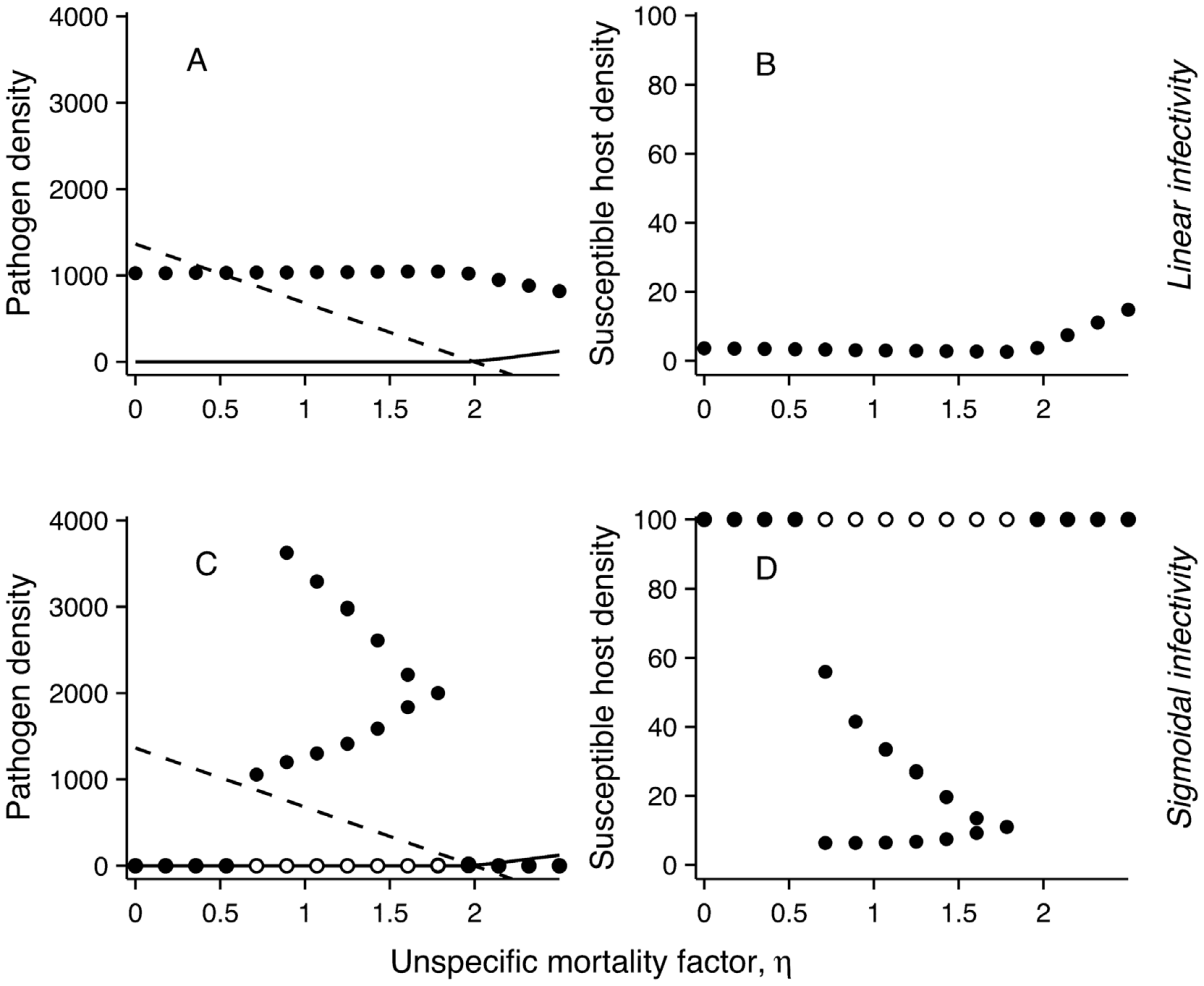
Density-independent mortality *η* affects pathogen and susceptible host densities. Here the number of competitors is fixed to 15 and pathogen competitive disadvantage α is fixed to 0.05. Linear infectivity response with rate constant *β*/2*ID*_50_ was used in panels (a) and (b), and sigmoidal response (eqn. 2) in panels (c) and (d). Filled symbols represent minimum and maximum densities. In the cyclic range in (c) and (d) open symbols show the alternative attractor and filled symbols show minima and maxima. The solid and dashed lines indicate the equilibrium pathogen and non-pathogenic competitor densities without hosts, respectively.

Increasing mortality (*η*) in outside-host environment reduces the competitive pressure from the rest of the outside-host community on the pathogen. When this density-independent mortality is intermediate, the pathogen can thrive in environments where competition would otherwise drive it to extinction (Fig. 4c). The reason for this is that increasing *η* reduces the density-dependent negative feedback from competitors to the pathogen, leading to better utilisation of the fitness benefits from within-host reproduction. This effect is not seen when the infectivity response is linear (Fig. 4a).

If the mortality term is taken as non-specific antibiotic treatment, the effect of increasing mortality can depend on initial conditions with the sigmoidal infectivity: If the pathogen is present in the system, sufficient antibiotic treatment leads to pathogen extinction. In contrast, if the pathogen is initially at a very low density and antibiotics are applied as a precautionary measure, the treatment can paradoxically result in pathogen outbreaks. The scenario, where *η* is equal for all environmental species, represents a limiting case along a continuum where the pathogen is affected by the antimicrobial substance either more or less than on the competing species on average. If the treatment targets the pathogen more than other species, the probability of an outbreak is reduced. In the worst case the treatment targets the pathogen less than the competitors leading to an increase in pathogen density by reducing competition.

## Discussion

Environmentally growing opportunists are common class of pathogens but there are few attempts to understand how environmental growth and ecological interactions outside the host affect epidemiological dynamics. We present here an analysis of the epidemiology of an environmental pathogen that has sustained growth in the absence of hosts and interacts with non-pathogenic organisms through competitive interactions. The results stress three important factors affecting the host–pathogen interaction: (1) The shape of the infectivity response has a strong impact on the dynamical behavior of the system; (2) Under a sigmoidal dose-dependent pathogen infectivity response reducing species richness of non-pathogenic competitors in the outside-host environment provides a novel mechanism for disease outbreaks; (3) With the sigmoidal infectivity too high pathogen virulence can lead to host extinction, which leads to virulence becoming useless for the pathogen.

### Microbial Diversity and Disease Dynamics

The importance of biodiversity on the stability and functioning of ecological communities continues to motivate ecological research [39]–[42]. A common observation is that increasing diversity tends to promote stability of community biomass [43], [44]. The importance of diversity in ecological systems and the ubiquitousness of environmentally growing pathogens are well known [8], but the theory connecting diversity to disease outbreaks is centred mainly on host diversity [45] with some work on pathogen diversity [46]. Our results show that the intensity of competitive interactions–modified either through community size or the strength of interspecific interactions–in outside-host environments can be very important for the dynamics of environmental opportunistic pathogens and the occurrence of disease outbreaks.

These results are in agreement with numerous empirical observations promoting the importance of biodiversity for disease dynamics. For example, biodiversity loss has been associated with both increases and decreases in disease transmission [47]. Loss of fungal diversity in agricultural soil has been shown to result in higher incidence of fungal plant diseases, and it is noteworthy that even generally non-pathogenic fungi can cause diseases if they are the predominant species [48]. Loss of bacterial diversity has been linked to problems in the human intestine such as inflammatory bowel diseases [49], and low microbial diversity is also suspected to be the cause of several allergies [50].

### The Role of the Pathogen’s Infectivity Response

Our results indicate that the way increasing competitive pressure on the pathogen in the outside-host community affects pathogen dynamics depends crucially on the shape of the pathogen’s infectivity response (i.e., how pathogen infectivity depends on its own density) (Figs. 3, S1). A common assumption in epidemiological models is that the relationship between pathogen number and infections is linear [30]. In this case the pathogen (suffering a cost in competitive ability) is able to compensate for reduced density due to competition via fitness benefits from host infection, and is relatively unaffected by varying, e.g., community size in the outside-host environment (Fig. 2a). This is because the pathogen remains infective even at low densities. In contrast, under a sigmoidal infectivity response that incorporates an infective dose [36], [51] increasing community size can either generate cyclic pathogen outbreaks, or drive the pathogen extinct (Fig. 2c). If pathogen densities are reduced sufficiently, the pathogen is unable to (re)enter the infection cycle.

Similarly to the direct host-to-host transmission of obligatory pathogens studied by Regoes et al. [36], the sigmoidal infectivity response is a disadvantage to the pathogen also in our model with environmental transmission and growth. This is due to the Allee effect associated with sigmoid response [36]. Increasing competitive pressure on the pathogen eventually leads to a situation where the pathogen is unable to recover from low densities and cause infections, resulting in pathogen extinction. On the other hand, when competition is weak the pathogen can drive the host extinct, where after infectiveness becomes useless to the pathogen. This phenomenon resembles the consequences of the classical trade-off between virulence and transmission in obligate pathogens [52], [53], but arises from a completely different mechanism. The host represents simply a resource for the pathogen that may in cases be ‘over-exploited’ and lost, reducing the system to the outside-host community. Over-exploitation of the host is the mechanism behind cyclic host-pathogen dynamics, similarly to the mechanism underlying cyclic consumer-resource dynamics [54].

As indicated above, the shape of the infectivity response at low pathogen densities has a profound impact on the dynamics. If infectivity is low (and increases slowly) at low pathogen densities, the pathogen needs to reach relatively high densities in the environment to become infective. This can in turn be prevented by, e.g., ecological interactions in the outside-host environment that reduce pathogen densities (such as competition, predation, and parasitism). The importance of the shape of infectivity response in environmental transmission has been recognized by Boldin & Kisdi [29] in evolutionary context. They argued that a concave infectivity response enables evolutionary branching. Here we have shown that convexity of the infectivity response at low pathogen densities can be very important for epidemiological dynamics of opportunistic, environmentally growing pathogens.

### Environmental Growth

Outside-host growth of pathogens is widespread in nature. Models of environmentally transmitted diseases typically allow only exponential decay of pathogens [10], [12] or in some cases density-independent growth [9], [51], [55], [56]. To our knowledge, density-dependent growth has been analysed only by Merikanto et al. [11]. Outside-host growth and the recovery of infected hosts have a strong stabilising influence on pathogen dynamics. Density dependent growth dampens the influence of pathogens shed from infected hosts, when the outside-host pathogen population is near its carrying capacity [11]. Recovery is stabilising because it moderates the decline of susceptible hosts after a disease outbreak.

Under a linear infectivity response density-dependent pathogen growth in the outside-host environment effectively filters the inflow pathogens from infected hosts, resulting in stable population dynamics [11]. Cyclic outbreaks can arise only when pathogens do not exhibit active growth in the outside-host environment (i.e., there is only an exponential decay of pathogen densities), and when the pathogen is extremely lethal. A sigmoidal infectivity response is more prone to generate cyclic population dynamics because after an outbreak host growth is more rapid than the increase in pathogens infectivity at low densities, allowing the host supply to recover before the next outbreak.

### Implications

Multi-cellular hosts are high resource environments with a potentially deadly immune system for parasites. Parasites often have to allocate considerable amount of resources for infecting and overcoming the immune system, and sacrifice part of their competitive ability in the outside host environment [38]. However, the payoff is that once the immune system is defeated the nutrient rich body may be consumed to gain massive fitness benefits. An example of an effective and highly virulent environmental pathogen that grows slowly in the environment is *Flavobacterium columnare*, a saprotrophic fish pathogen that causes considerable economical losses in fisheries [17]. Other examples of well-studied environmental saprotrophic pathogens are *Serratia marcescens* and *Pseudomonas sp*. both capable of infecting a wide range of hosts.

Opportunism coupled with the ability to grow in the free-living environment, may be an important step in the evolution of virulence for bacteria. This naturally requires that pathogens are released from the infected host to the environment, i.e., the host is not an ecological or evolutionary dead end. Free-living bacteria may develop infectivity but lack the means for effective host-to-host transmission. Thus they would benefit from infectivity only if they are virulent enough to gain reproductive output from the host and survive in the environment until encountering a new host. The waiting time before a new infection may be long and the costs of maintaining infectivity traits are likely to restrict the growth rate of the pathogen. If the resources are sparse and there is competition for them, the non-pathogenic competitors are likely to out-compete the pathogen, and this may lead to local pathogen extinction.

Applying a non-specific mortality factor upon the outside-host community can reduce competitive pressure on the pathogen. While the density of all species, including the pathogen, is reduced equally in the outside-host environment, the pathogen gets an indirect advantage through within-host reproduction (Fig. 3). This suggests that the use of antimicrobial substances as a means of controlling pathogen growth does not always have the desired effect. If all species in the outside-host community are similarly susceptible to the substance, its action is reasonably modeled as a linear mortality term imposed on all members of the outside-host community. This can in some cases help the pathogen by removing competition. It would then be crucial to use enough antimicrobial substances to reach the range where the pathogen cannot survive. It would also be possible that the introduction of effective non-pathogenic competitors could work as a defense measure in such conditions because treatment of hosts in environmentally growing pathogens is ineffective [11], [51].

## Supporting Information

Figure S1 **Equilibrium pathogen and susceptible host densities as a function of competitive disadvantage of the pathogen (α).** Black dots represent mean densities. At cyclic ranges open circles represent an alternative attractor and filled symbols indicate minima and maxima. Here the number of competitors is fixed to *n = *7 and linear mortality parameter *η* to 1.0. Linear infectivity response with rate constant *β*/2ID_50_ was used in panels (A) and (B), and sigmoidal response (eqn. 2) in panels (C) and (D). The solid line is the equilibrium pathogen density without hosts.
(TIF)

Figure S2 **Mean pathogen densities versus competitive disadvantages of the pathogen (α) from sensitivity analysis replicates.** In panel (A) with linear infectivity response red dots represent individual simulation outcomes. In panel (B) with sigmoidal infectivity response cyan dots represent outcomes from simulations resulting in stable dynamics (s.d.(*P*) <10). Red and blue dots represent outcomes from simulations resulting in cyclic dynamics (s.d.(*P*) >10). To distinguish between alternative outcomes, values above 1000 are coloured red and those below 1000 are blue. Grey dots represent standard deviations (s.d.(*P*)) above and below the mean value. The replicates resulting in host extinction (mean(*S*) <5) have been excluded from both panels. Panel (A) has 50720 points and panel (B) has 86615 coloured points of which 14159 are cyclic and 72456 are stable. Total number of simulations is 100000 in both cases.
(TIF)

Figure S3 **Posterior parameter distributions from sensitivity analysis replicates not resulting in host extinction (mean(*S*) >5)**. The parameter values for unspecific mortality rate (*η*), host growth rate (*r_h_*), host recovery rate (*δ*), infected death rate (*ν*), and pathogen release (*k*) were picked from uniform random distributions. Selecting the cases with host persistence resulted in 50720 replicates in the upper panels (linear infectivity) and 86615 replicates in the lower panels (sigmoidal infectivity) from a total of 100000 replicates for each infectivity response.
(TIF)

Materials S1 **Sensitivity analysis.**
(DOCX)
