# Supplementary figures and images for: Loss of Competition in the Outside Host Environment Generates Outbreaks of Environmental Opportunist Pathogens
Source: PLoS One. 2013 Aug 16;8(8):e71621. doi: 10.1371/journal.pone.0071621 (PMC3752018; doi:10.1371/journal.pone.0071621)

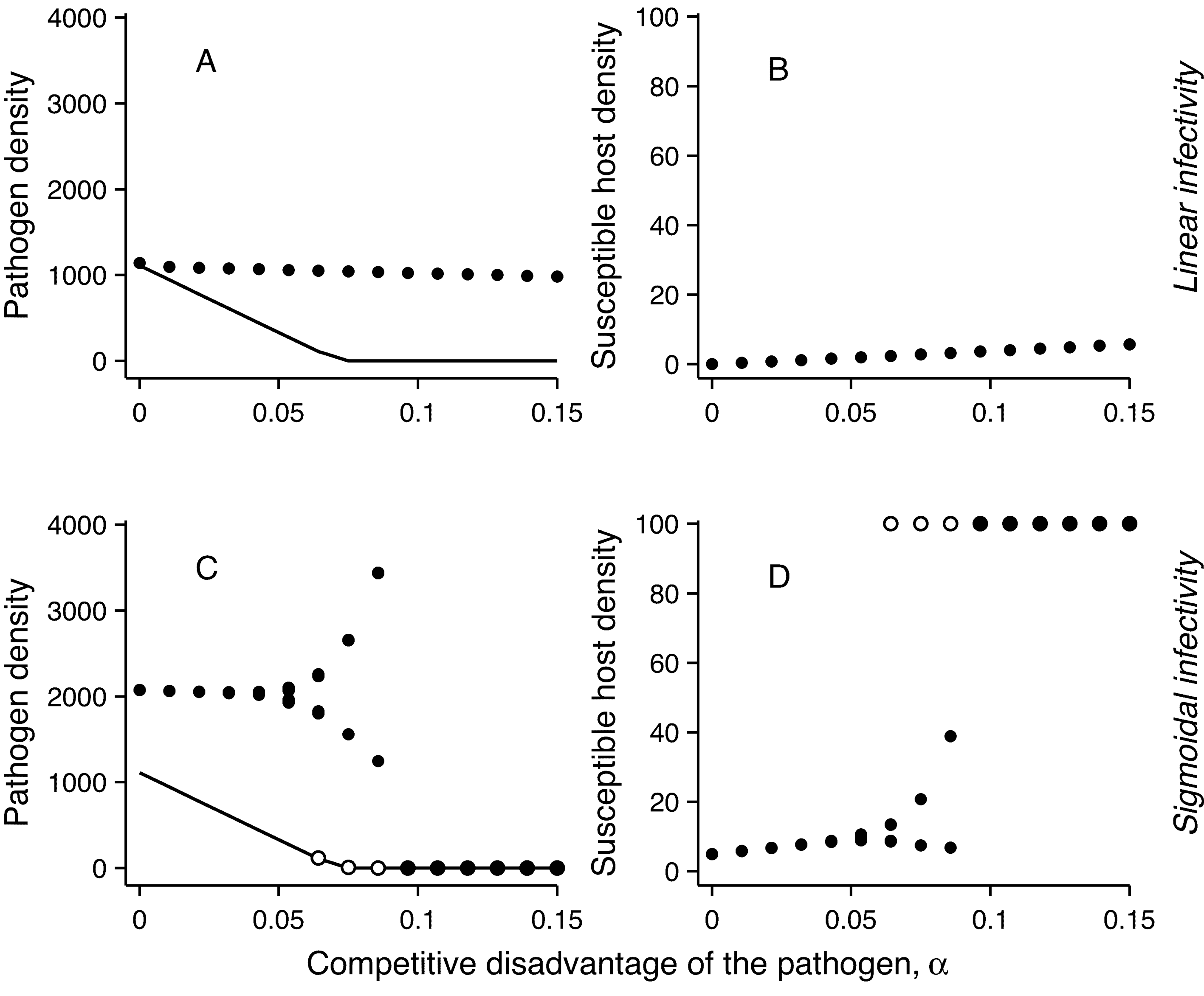

Supplement: Figure S1 — Equilibrium pathogen and susceptible host densities as a function of competitive disadvantage of the pathogen (α). Black dots represent mean densities. At cyclic ranges open circles represent an alternative attractor and filled symbols indicate minima and maxima. Here the number of competitors is fixed to n = 7 and linear mortality parameter η to 1.0. Linear infectivity response with rate constant β/2ID50 was used in panels (A) and (B), and sigmoidal response (eqn. 2) in panels (C) and (D). The solid line is the equilibrium pathogen density without hosts. (TIF) [file pone.0071621.s001.tif]

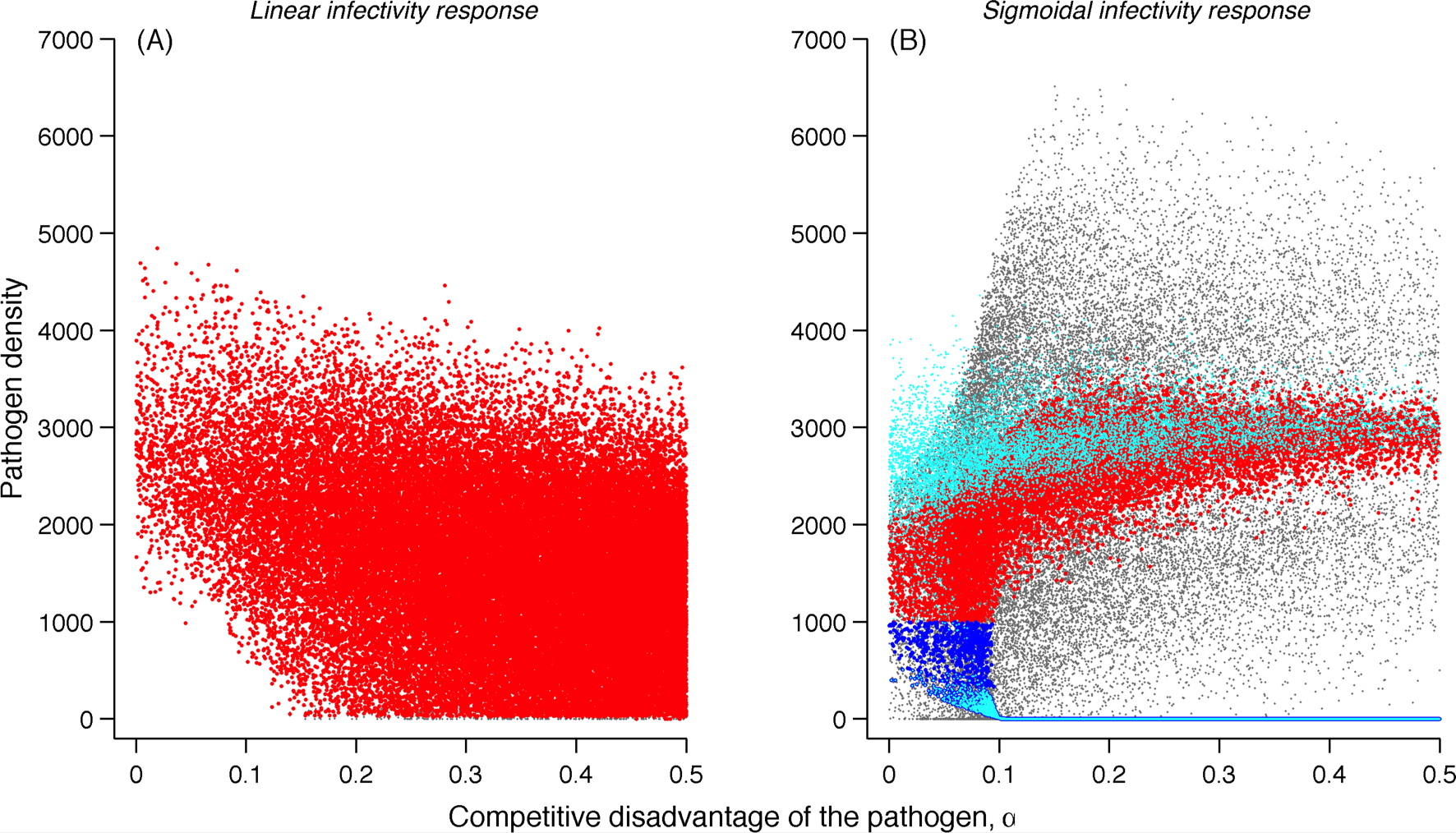

Supplement: Figure S2 — Mean pathogen densities versus competitive disadvantages of the pathogen (α) from sensitivity analysis replicates. In panel (A) with linear infectivity response red dots represent individual simulation outcomes. In panel (B) with sigmoidal infectivity response cyan dots represent outcomes from simulations resulting in stable dynamics (s.d.(P) <10). Red and blue dots represent outcomes from simulations resulting in cyclic dynamics (s.d.(P) >10). To distinguish between alternative outcomes, values above 1000 are coloured red and those below 1000 are blue. Grey dots represent standard deviations (s.d.(P)) above and below the mean value. The replicates resulting in host extinction (mean(S) <5) have been excluded from both panels. Panel (A) has 50720 points and panel (B) has 86615 coloured points of which 14159 are cyclic and 72456 are stable. Total number of simulations is 100000 in both cases. (TIF) [file pone.0071621.s002.tif]

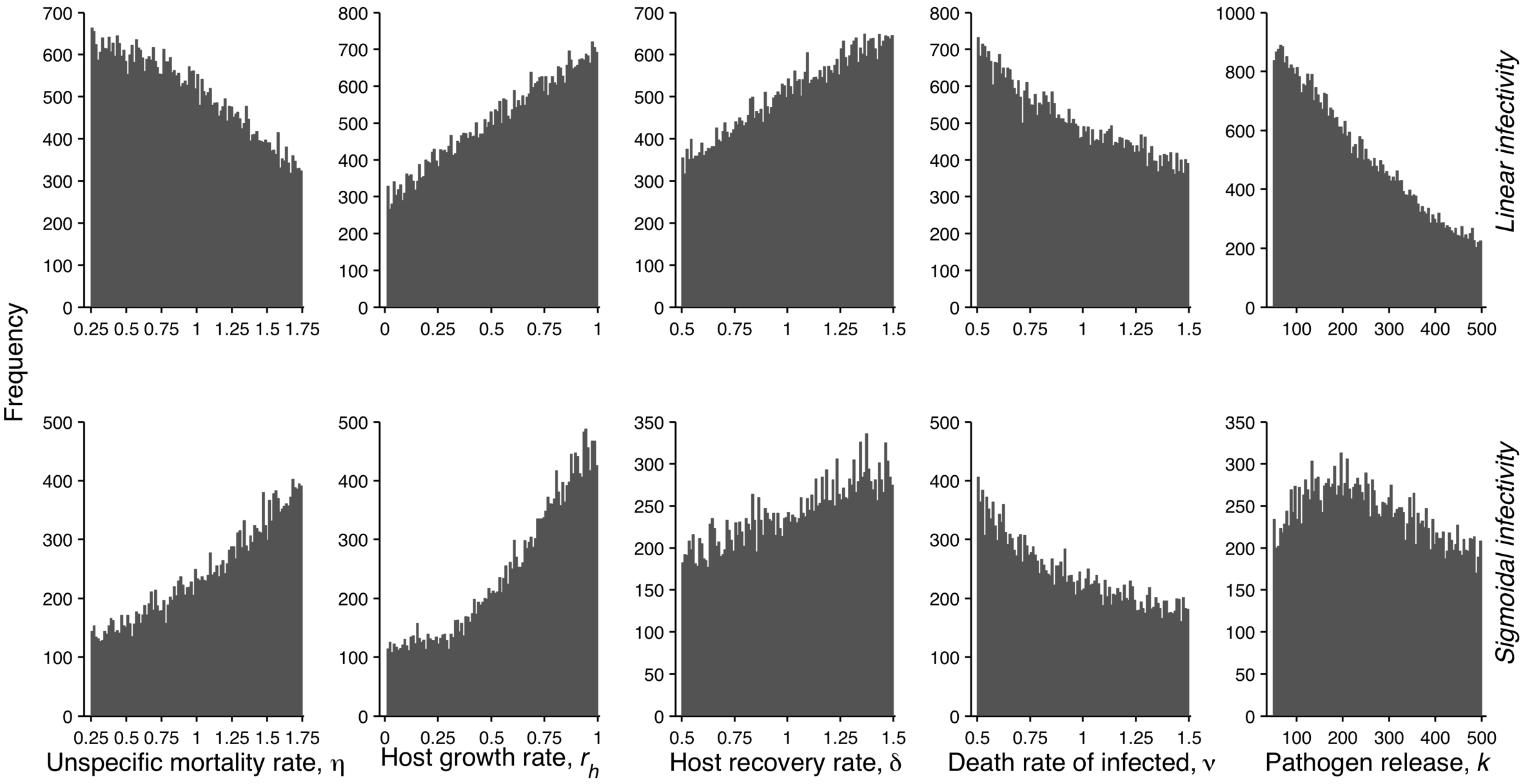

Supplement: Figure S3 — Posterior parameter distributions from sensitivity analysis replicates not resulting in host extinction (mean(S) >5). The parameter values for unspecific mortality rate (η), host growth rate (rh), host recovery rate (δ), infected death rate (ν), and pathogen release (k) were picked from uniform random distributions. Selecting the cases with host persistence resulted in 50720 replicates in the upper panels (linear infectivity) and 86615 replicates in the lower panels (sigmoidal infectivity) from a total of 100000 replicates for each infectivity response. (TIF) [file pone.0071621.s003.tif]
