# Supplementary material for: Loss of Competition in the Outside Host Environment Generates Outbreaks of Environmental Opportunist Pathogens
Source: PLoS One. 2013 Aug 16;8(8):e71621. doi: 10.1371/journal.pone.0071621 (PMC3752018; doi:10.1371/journal.pone.0071621)
Supplement: Materials S1 — Sensitivity analysis. (DOCX) [file pone.0071621.s004.docx]

# SUPPORTING INFORMATION

## Sensitivity analysis

The sensitivity of the different dynamical outcomes to the parameter values was tested by repeating the simulation 100 000 times with randomly chosen parameter values for host growth rate *r_h_* between [0.01,1.0], number of pathogens released per killed host *k* [50,500], recovery rate *δ* [0.5,1.5], infection kill rate ν [0.5,1.5], linear mortality rate *η* [0.25,1.75], and reduction in competitive ability *α* [0,0.5] from the uniform distribution. The size of the outside community *S* was fixed to 6 species. The rest of the parameters were set to the values in table 1 in main text. This procedure was done separately for the sigmoidal and linear infectivity responses.

The randomization results for linear infectivity response, assuming host persistence, are in agreement with those given in Figure S2A for the specific parameter set used in the main text; pathogen densities decrease under increasing competitive disadvantage (α). The results for sigmoidal infectivity response are also in good qualitative agreement with those shown in Figure S2C. For example, the randomization experiment predicts a similar existence of alternative states for the pathogen under relatively high values of α (Figure S3B). Also, for parameter combinations associated with cyclic pathogen outbreaks, there is an increase in the cycle amplitude with increasing α as observed for the specific parameter set in Figure S2C. The non-cyclic combinations are due to host growth rate being too high or too low in relation to other parameters (Figure 2, main text). Finally, the probability of pathogen persistence is reduced considerably under relatively high α (Figure S3B).

The different infectivity responses have relatively similar selection effects for posterior parameter distributions when replicates resulting in host extinction are excluded (Figure S3) with the exception of linear mortality rate (𝜂). For the linear infectivity response the result is in accordance with the slight decrease in host densities under increasing 𝜂 as observed in Figure 3 in main text. For the sigmoidal infectivity response the increase in host survival under increasing 𝜂 is explained by an increase in the probability of pathogen extinction.
